# The Effect of Hf Addition on the Boronizing and Siliciding Behavior of CoCrFeNi High Entropy Alloys

**DOI:** 10.3390/ma15062282

**Published:** 2022-03-19

**Authors:** Sezgin Cengiz, Mattias Thuvander

**Affiliations:** 1 Department of Materials Science and Engineering, Gebze Technical University, Gebze 41400, Turkey; 2 Department of Physics, Chalmers University of Technology, 412 96 Göteborg, Sweden

**Keywords:** high entropy alloy, boronizing, Laves phase, surface hardening, boriding

## Abstract

The effect of a boronizing and siliciding process on CoCrFeNiHf_0.1–0.42_ high entropy alloys was examined in this study. When increasing the amount of added Hf in CoCrFeNiHf_x_, the structure of the alloys gradually transformed from single-phase FCC to firstly Ni_7_Hf_2_ + FCC, and finally to C15 Laves and FCC phases. The boronizing/siliciding process resulted in the formation of a silicon-rich layer and a boride layer (BL). Increasing the amount of Hf in the alloys resulted in a decrease in the combined layer thickness, which was measured for CoCrFeNi, CoCrFeNiHf_0.1_, CoCrFeNiHf_0.2_, and CoCrFeNiHf_0.42_ to be 70 µm, 63 µm, 20 µm, and 15 µm, respectively. In contrast, the thickness of the transition zone/diffusion zone increased with more Hf in the alloys. While silicon atoms were gathered close to the BL, they were not transferred into the CoCrFeNi substrate. In contrast to the observation for CoCrFeNi, Si atoms penetrated through the Ni-rich phase (Ni_7_Hf_2_) in the CoCrFeNiHf_x_ alloys. Furthermore, the Cr-B rich area (Cr_5_B_3_) in the coating limited the transport of Si into the CoCrFeNiHf_x_ substrates. XRD analysis showed that the BL contained Ni_2_Si, FeB, Fe_2_B, Co_2_B, and Cr_5_B_3_ phases.

## 1. Introduction

Recently, high entropy alloys (HEAs) have attracted the interest of the scientific community. They contain multiple basic alloy elements and have a wide composition range, which is attractive for the design of new alloys [1,2,3]. HEAs are defined as alloys with five and more metallic elements, each of which has an atomic percentage between 5% and 35% [1,2,3]. However, single-phase HEAs have been reported to often not fulfill the high requirements on mechanical properties. When atoms of different sizes and/or low negative enthalpy are added to single-phase CoCrFeNi alloys, new multiphase alloys are formed [1,2,3,4,5,6,7,8,9,10]. They have unique compositions and microstructures, and they exhibit attractive properties, including high strength, hardness, wear resistance, high temperature strength, good oxidation resistance, and good structural stability for high temperature applications [1,2,3,4,5,6,11,12,13]. The excellent mechanical and chemical properties are provided by the intermetallic compounds present. The added different elements form new phases, such as Laves phases, which are the largest class of intermetallic structures [14,15]. Laves phases are topologically close-packed (TCP) phases with ideal composition AB_2_ (B is the small atom and A is the large atom) that crystallize with the cubic C15 (MgCu_2_) structure or the hexagonal polymorphs C14 (MgZn_2_) and C36 (MgNi_2_) [15,16,17]. In Laves structures, the A atoms take up ordered positions as in diamond, while the B atoms take up tetrahedral positions around the A atoms. In case the atomic size ratio of A and B is about 1.225, they can form TCP structures with an overall packing density of 0.71 [18]. The Laves phases can significantly improve the mechanical and thermal properties of alloys [4,5,14,15,17].

HEAs containing Laves phases display high temperature stability and excellent mechanical properties of the bulk, but the surface properties of such alloys restrict their applicability [19,20,21,22,23,24]. Laves phases consist of different elements such as Nb and Hf. Small amounts of Nb and Hf are often added to steel and Ni alloys to improve oxidation resistance and it could be expected that these elements should have a similar effect on HEAs. However, results in the literature suggest that the approach was not successful. The high temperature oxidation behavior (at 700, 800, and 900 °C for 100 h) of CoCrFeMnNb_x_Ni (x = 0 and 0.25) HEAs in air was investigated [25]. CoCrFeMnNb_0.25_Ni alloy consisting of FCC + Laves phase exhibited that Laves phase in the substrate at the oxide/alloy interface could hinder the outward diffusion of metal ions and improve the oxidation resistance (700 and 800 °C). Nonetheless, Laves phase in substrates can facilitate the inward diffusion of oxygen ions and result in internal oxidation behavior (900 °C) [25]. In another study, AlCoCrFeNi HEA showed a high oxidation rate and a low spallation resistance [26]. The oxidation behavior of HEAs can be enhanced by using minor reactive elements such as Y (0.02 at. %) and Hf (0.02 at. %). Such additions into AlCoCrFeNi led to a significantly lower oxidation rate and a strong oxide spallation resistance (1100 °C) [26]. In contrast, Hf-doped Ni alloys displayed that the growth rate of the oxide scale decreases when the Hf addition is 0.02 at. %. Then, with increasing content of Hf from 0.02 to 0.09 at. %, the growth rate of the oxide scale increased. Consequently, it was stated that there is an increase in spallation resistance when the amount of Hf increases from 0 to 0.02%, and then this tendency decreases when the addition of Hf increases from 0.02% to 0.9% [27]. According to these results, the addition of Hf to CoCrFeNi is reported to favor the formation of Ni_7_Hf_2_ and Laves-C15 phase due to the high mixing enthalpies of Co, Cr, Fe, and Ni with Hf. The new phases are rich in Ni and Hf. It has been shown that a high concentration of Hf and Ni in HEAs does not affect the oxidation resistance positively. Therefore, alternative methods are highly needed to improve the high temperature application of Hf-doped HEAs. Hence, various surface modification methods have been applied to enhance the surface properties of HEAs. Protective layers have been synthesized using uncomplicated processes, including boronizing, nitriding, carburizing, and siliciding [19,20,21,22,23,24,28]. The boronizing and siliciding processes are thermo-chemical surface treatment methods, similar to nitriding and carburizing. A boride or a silicide layer on the workpiece improves the mechanical and chemical properties of the surface. In these methods, the diffusion and reaction of boron and silicon atoms produce boride/silicide layers at the surface of the substrates and into the metal.

Recent studies have shown that the growth, morphology, thickness, phase composition, and hardness of the formed boride layer are strongly dependent on the alloying elements and the microstructure [19,20,21,23,29,30,31,32,33,34,35,36,37]. Iron-based alloys have shown diverse boronizing behaviors since the alloying elements may diffuse into the boride coating. Similarly, the chemical composition of HEAs is complex and contains a wide variety of alloying elements. Therefore, the influence of an individual alloying element on the boronizing process is not easy to explain due to the diversity of the effects of every individual element on the boride layer properties. The equiatomic manganese doping to CoCrFeNi alloys has caused remarkable solid solution, and single FCC phase, which exhibited the same thickness and the same morphology in the boride layer for CoCrFeNi and Mn-doped CoCrFeNi alloys [22]. The addition of equiatomic Ti and Nb elements with a large difference in enthalpy of mixing and atom size caused significant differences in FCC-CoCrFeNi alloys. The thickness of the BL decreased with the amount of added Ti and Nb [19]. Günen et al. investigated the effect of surface treatment temperatures (900, 950, and 1000 °C) on Co_1.19_Cr_1.86_Fe_1.30_Mn_1.39_Ni_1.05_Al_0.17_B_0.04_ using nanosized B_4_C (Si-free powder mixture). The layer on the boronized alloys consisted of complex phases (such as Cr_2_Ni_3_B_6_, (Fe_0.4_Mn_0.6_)B, and, (Cr_0.4_Mn_0.6_)B). The hardness values of the boronized alloys increased by 7–10 times. (The hardness of as-cast and borided layers were 235–260 HV and 1691–2730 HV, respectively.) The boride layers on the alloys decreased the coefficient of friction and the wear resistance with increasing surface treatment temperature [38]. It was reported that a self-lubricating coating was developed with the electrochemical boronizing process. The hardness of the boronized alloy was about 2130 HV, which was almost four times higher than for the as-cast AlCoCrFeNi_2.1_ alloy. (The hardness of the as-cast alloy was nearly 530 HV.) The wear rate of boronized AlCoCrFeNi_2.1_ alloy was roughly 80% lower than that of as-cast alloy in water [39]. In another work, the tribological properties of boronized Fe_40_Mn_20_Cr_20_Ni_20_ alloys were investigated under different conditions (air, deionized water, and seawater). The hardness values were enhanced from 140 HV (hot-rolled alloy) to 970 HV (boronized alloys). The wear rate decreased under the different conditions and it was stated that the wear mechanisms differed depending on the test environment [40]. Although borided layers show excellent properties, especially in tribological applications, they may not show the same success in high temperature corrosion/oxidation. At moderate temperatures (it is about 1000 °C), a boric oxide (B_2_O_3_) glass layer forms on the boride layer. With increasing temperature, the boric oxide layer starts to evaporate and the protective layer against the aggressive external environment disappears [41]. When SiC is added to the system, it can improve the oxidation resistance of the boride layer. The formation of a silica-containing oxide layer is promoted with high temperature and it results in that the silica-based glass can offer greater resistance than B_2_O_3_ alone [41,42,43]. It is well-known that silicide-based structures are attractive to exhibit excellent wear, corrosion, and oxidation resistance in aggressive and complex environments (high temperature, atmosphere containing oxygen, sulfur). Similar to Al_2_O_3_, SiO_2_ is the most protective structure owing to its high thermodynamic stability and low diffusivity for ions [44,45]. Nonetheless, the siliciding process is avoided during the boriding of nickel alloys and different mixed powders (without SiC powder) are used during boronizing of pure nickel and nickel-based alloys. For the silicide-based coatings, the interaction between the layer and the substrate is very fast during high temperature applications, this is a disadvantage for these coatings. Multi-layered structures may be the best approach to solving these problems [46,47,48].

The aim of this study was to investigate the effects of the alloying element hafnium and/or the Laves phase (C15 Laves) content systematically on the boronizing behavior of CoCrFeNi, and in this study, an attempt was made to understand the boronizing and siliciding mechanism on the CoCrFeNiHf_x_ alloys by increasing the Hf content step by step. It has been shown that the formation of Laves phase, either as a major or a minor phase, depends on the amount of the added alloying element. For this purpose, CoCrFeNiHf_x_ alloys containing a wide range of Hf (x = 0.1, 0.2, 0.42) were prepared by using a vacuum arc melter system. These compositions were chosen deliberately, and the CoCrFeNiHf_0.42_ alloy is at the eutectic point. This alloy was named the eutectic high entropy alloy (EHEA) and showed the formation of typical eutectic or lamellar microstructures. The borided samples were characterized using X-ray Diffraction (XRD) and Scanning Electron Microscopy (SEM) together with Energy Dispersive Spectroscopy (EDS).

## 2. Experimental

Before the preparation of the CoCrFeNi and CoCrFeNiHfx (x = 0.1, 0.2, 0.42) alloys by arc melting, elemental pieces were weighed and mixed according to the respective composition and, the total weight of each alloy was 25 g. The alloys were prepared by using high purity elements in an atmosphere-controlled vacuum arc melter system (2.1 × 10^–6^ mbar) and under an argon atmosphere (700 mbar). The arc melting was performed in a water-cooled copper mold. The alloy ingots were re-melted five times to turn them into a homogeneous mixture. The alloys were cut into the dimensions of 10 × 10 × 2.5 mm^3^ using a low-speed saw. The surface of the samples was ground sequentially using 240–2500 grit SiC abrasive papers and then polished using alumina solution (Buehler, Lake Bluff, IL, USA, 0.05 µm). Finally, the samples were washed with water and ethanol in an ultrasonic bath for 5 min. The surface of the alloys was etched to determine the phases of the alloys. The etching process was applied by dipping into the etching solution. The microstructure and elemental examination of the fabricated alloys were conducted using an FEI Quanta 200 FEG ESEM (Thermo Fisher Scientific, Waltham, MA, United States) equipped with an EDS detector (Oxford Instrument, Oxfordshire, UK). For the boronizing/siliciding treatment, the alloys were placed in an alumina crucible and packed with Ekabor II™ powder composed of 90% SiC, 5% B_4_C, and 5% KBF. Fine Ekrit™ powder and fine alumina powder were added to the Ekabor II as a cover layer to prevent oxidation during the process. The process was carried out for 5 h at 1000 °C in an electrical box furnace. Then, the samples were cooled to room temperature in air. The borided samples were brushed and then cleaned with ethanol. XRD analysis of the borided alloys was applied with Cu-K_α_ radiation (λ = 1.5406 Å) using a Bruker Advance D8 Diffractometer (Bruker, Billerica, MA, USA) (40 kW, 40 mA) with a 2θ range of 20–80° (scan rate of 1°/min). The samples were cut in the middle to reveal the cross-section. For investigating the microstructure of the alloys, samples were prepared by epoxy resin mounting, grinding (240–2500 grit SiC abrasive papers), and polishing (alumina polishing suspension—Buehler, 0.05 µm). The microstructure and chemical composition examination of the borided alloys were conducted using SEM/EDS (Oxford Instrument, Oxfordshire, UK).

## 3. Results

**Figure 1:**
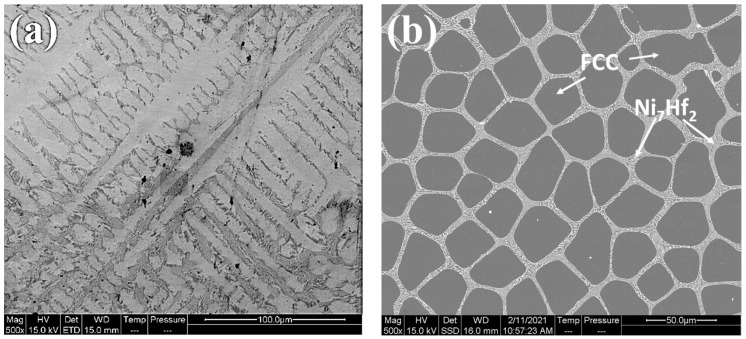

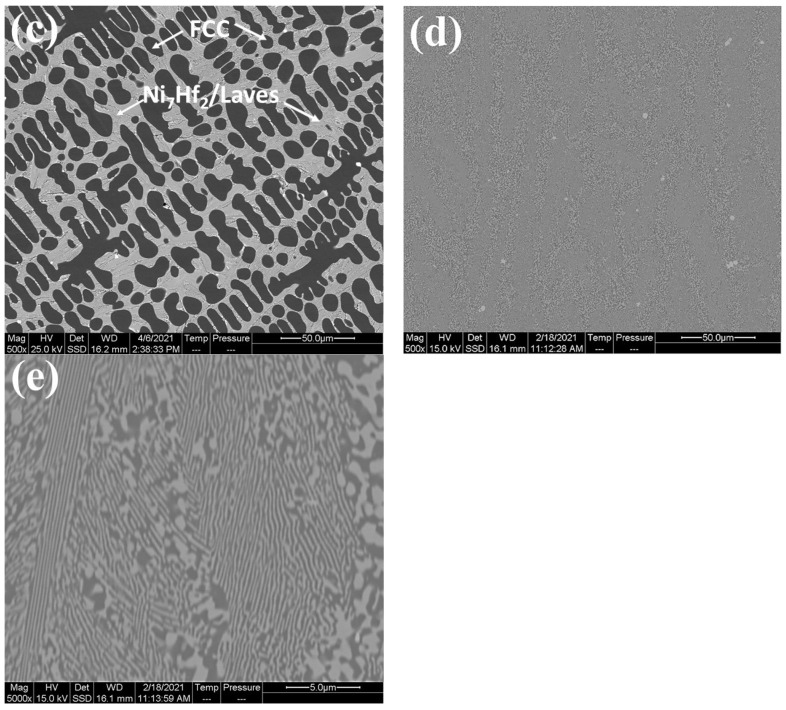
SEM micrographs of CoCrFeNi and CoCrFeNiHf_x_ alloy, (**a**) CoCrFeNi, (**b**) CoCrFeNiHf_0.1_, (**c**) CoCrFeNiHf_0.2_, (**d**,**e**) CoCrFeNiHf_0.42_. Note that the magnification is higher in (**e**).

**Figure 2:**
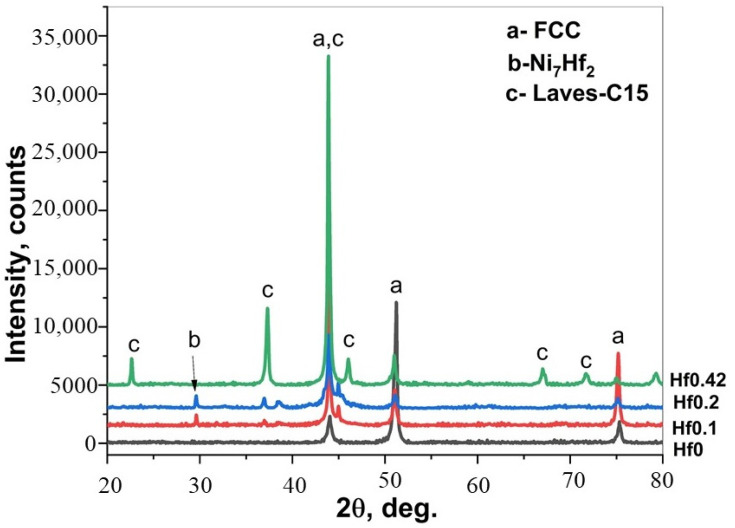
XRD scans of as-cast CoCrFeNi: Hf0, CoCrFeNiHf_0.1_: Hf0.1, CoCrFeNiHf_0.2_: Hf0.2, and CoCrFeNiHf_0.42_: Hf0.42.

Figure 1 and Figure 2, and Table S1, show the effect of Hf addition on the microstructure, phase constituents, and the elemental distribution of the CoCrFeNiHf_x_ HEAs before boronizing/siliciding. The quaternary CoCrFeNi medium entropy alloy was single-phase FCC, in accordance with previous studies [5,6]. The results of microstructure and phase analyses with increasing Hf content displayed that the structures of the HEAs gradually changed from single-phase FCC to, firstly, FCC + Ni_7_Hf_2_ and finally, FCC + C15 Laves. When the amount of Hf is increased, the volume fraction of the FCC phase diminishes, while the opposite holds for Laves. The alloy CoCrFeNiHf_0.42_ has a eutectic composition (Figure 1), as has been reported in other studies [5,6]. The Ni_7_Hf_2_/Laves phase in CoCrFeNiHf_0.1_ and CoCrFeNiHf_0.2_ have lamellar eutectic structures. When the content of Hf increases to approximately 10 at. %, the CoCrFeNiHf_0.42_ alloy consists partially of lamellar eutectic structure with parallel aligned lamellae (Figure 1d,e). While the FCC phase was rich in Cr, Co, and Fe, the Laves phase consisted of Ni and Hf (Table S1 and Figure 2).

**Figure 3:**
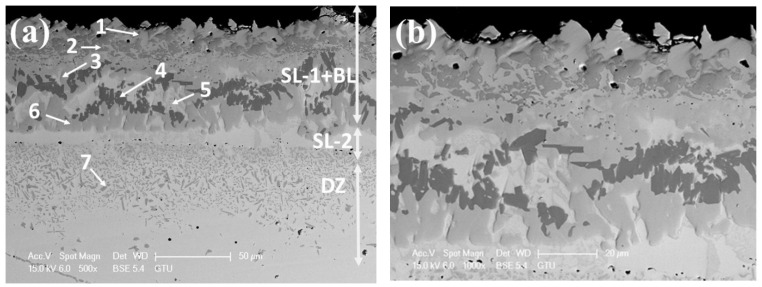

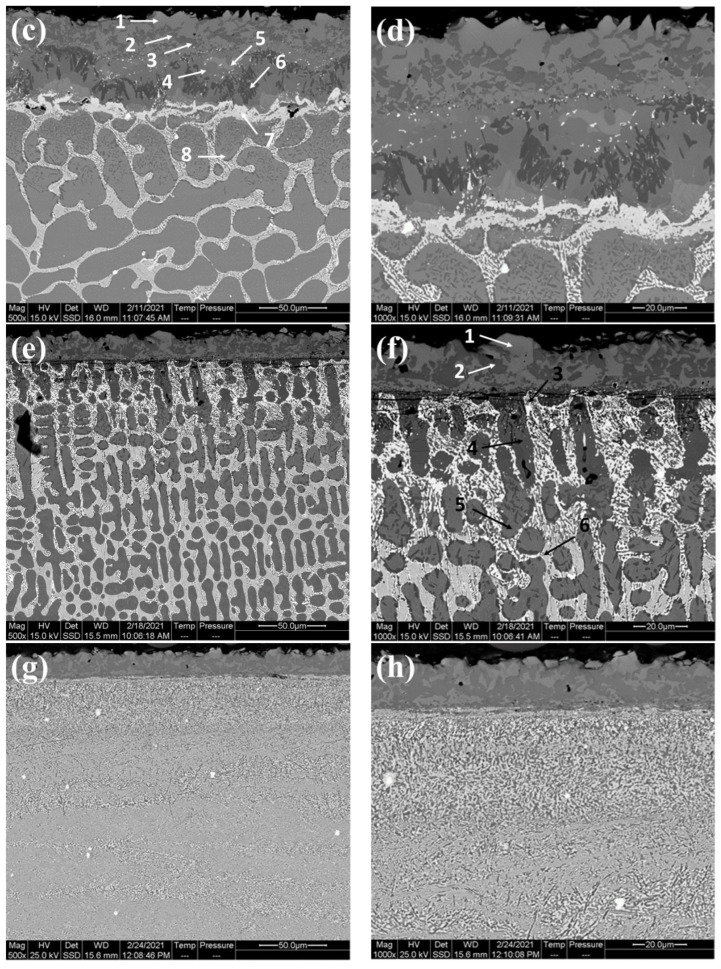
Cross-sectional SEM micrographs of borided CoCrFeNi and CoCrFeNiHf_x_, (**a**,**b**) CoCrFeNi, (**c**,**d**) CoCrFeNiHf_0.1_, (**e**,**f**) CoCrFeNiHf_0.2_, (**g**,**h**) CoCrFeNiHf_0.42_. The numbers in the images represent positions for spot EDS analysis and the EDS results are given in Table 1. Si-rich layer (SL-1), boride layer (BL), second Si-rich layer (SL-2), and transition zone or diffusion zone (DZ).

**Figure 4:**
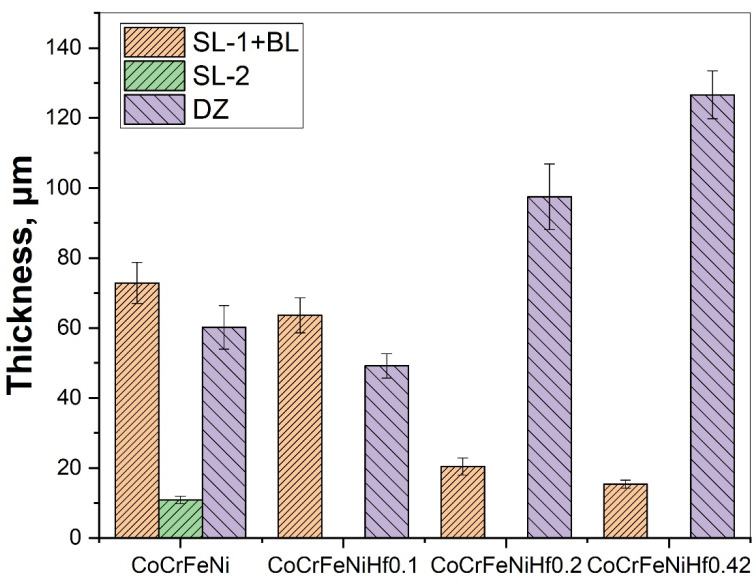
The thickness of layers for borided HEAs for varying Hf content. Si-rich layer (SL-1), boride layer (BL), second Si-rich layer (SL-2), and transition zone or diffusion zone (DZ).

Figure 3 shows microstructure images of the surface region of the four alloys, at different magnifications taken from the cross-sections of the samples after the boronizing/siliciding process. A Si-rich layer (SL-1), a BL, a second Si-rich layer (SL-2), and a diffusion zone (DZ) were identified in the CoCrFeNi alloys in Figure 3a,b and Figure S1. A more detailed microstructure of the BL on CoCrFeNi is shown in Supplemental Figure S1. Figure 4 shows that the thickness of the SL-1 + BL, SL-2, and DZ were approximately 72 ± 4 μm, 10 ± 1, and 60 ± 4, respectively.

**Figure 5:**
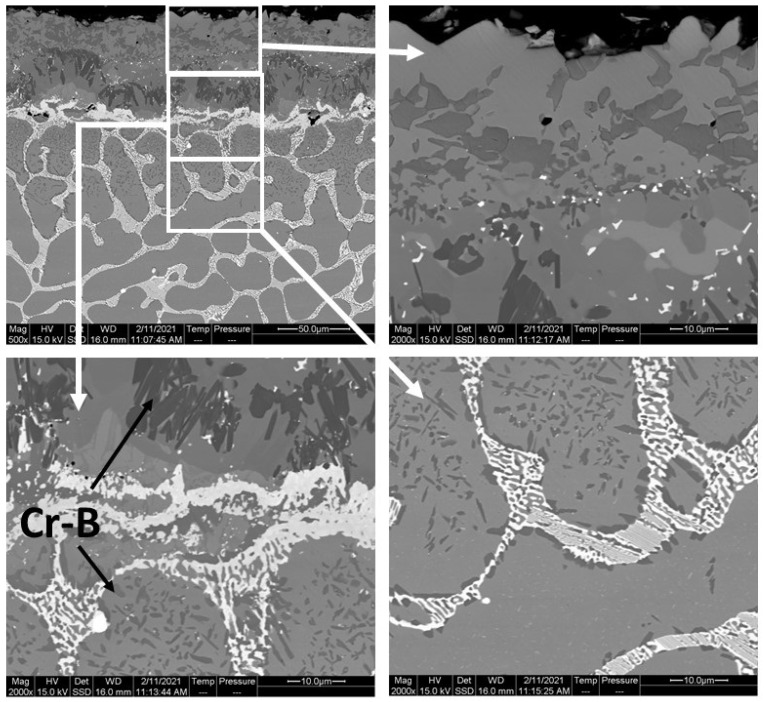
Cross-sectional SEM micrographs with different magnifications and regions of the borided CoCrFeNiHf_0.1_ alloy (the dark precipitates, marked by arrows, are rich in Cr and B).

**Figure 6:**
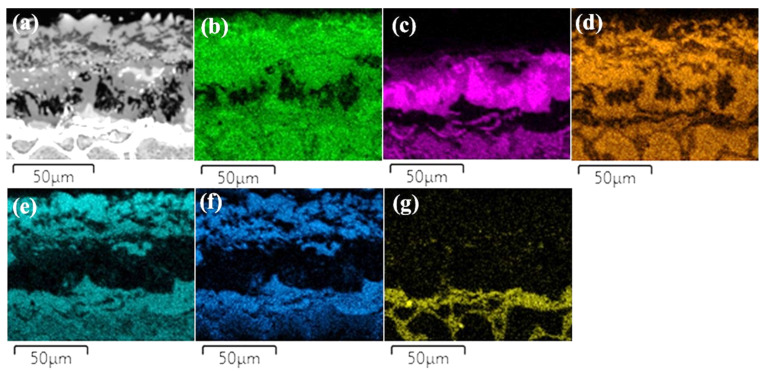
(**a**) SEM image of borided-CoCrFeNiHf_0.1_ and EDS elemental distribution maps, (**b**) Co, (**c**) Cr, (**d**) Fe, (**e**) Ni, (**f**) Si, (**g**) Hf.

**Figure 7:**
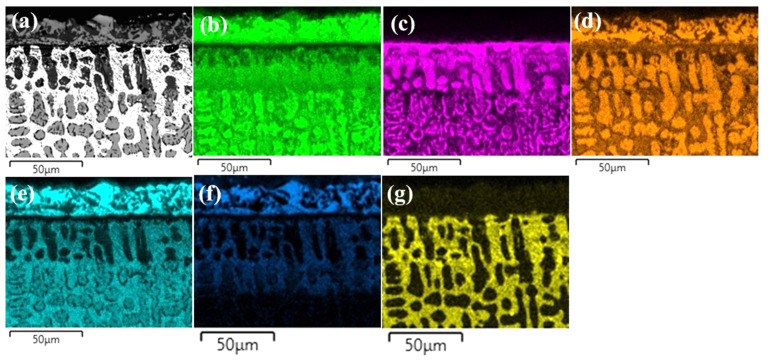
(**a**) SEM image of borided-CoCrFeNiHf_0.2_ and EDS elemental distribution maps, (**b**) Co, (**c**) Cr, (**d**) Fe, (**e**) Ni, (**f**) Si, (**g**) Hf.

**Figure 8:**
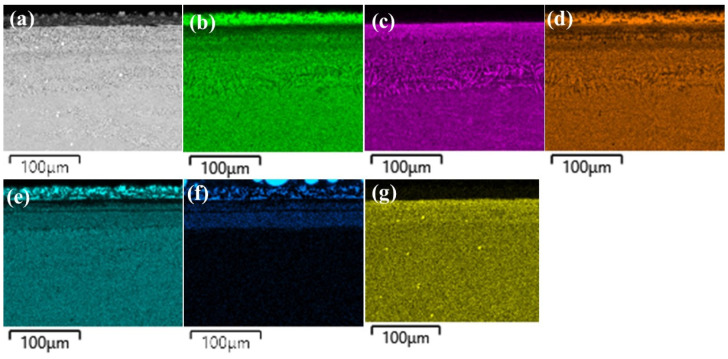
(**a**) SEM image of borided-CoCrFeNiHf_0.42_ and EDS elemental distribution maps, (**b**) Co, (**c**) Cr, (**d**) Fe, (**e**) Ni, (**f**) Si, (**g**) Hf.

The layers SL-1 + BL, and DZ were identified in the cross-sectional images of the borided alloys, see Figure 3, Figure 4, Figure 5, Figure 6, Figure 7 and Figure 8. Figure 4 shows that the thickness of SL-1 + BL and DZ were 64 ± 5 µm, and 50 ± 3.5 µm, respectively, for CoCrFeNiHf_0.1_. This figure further shows that the thickness of SL-1 + BL and DZ were 20 ± 2 µm, and 97 ± 9 µm, for CoCrFeNiHf_0.2_, and the thickness of SL-1 + BL and DZ were 15 ± 1 µm, and 126 ± 6 µm, for CoCrFeNiHf_0.42_, respectively. The interface between the SL-1 + BL and the substrate changed to a relatively smooth and compact appearance with the addition of Hf in CoCrFeNiHf_0.1_. The thickness and microstructure of the SL-1 + BL of CoCrFeNiHf_0.1_ were similar to the borided layer of CoCrFeNi. However, when high amounts of Hf were added, the thickness of SL-1 + BL decreased and the thickness of DZ increased. The cross-sectional SEM images with different magnifications of borided CoCrFeNiHf_0.1_ are presented in Figure 5. Dark precipitates are present in the SL-1 + BL and DZ. In addition, these precipitates were found to be Cr-B rich (Table 1), irregularly shaped, and roughly dispersed in the coating while being finely dispersed in the DZ for the borided alloys. SEM microstructure images of the CoCrFeNiHf_x_ alloys taken from the cross-section of the coating and elemental mapping are presented in Figure 6, Figure 7 and Figure 8. The EDS mapping of the borided alloys demonstrated that Co, Fe, and Ni were the dominating elements of the coating. Nevertheless, while Co and Fe appeared to be homogeneous in the coatings, Ni and Si appeared frequently at the outer part of the coatings. Furthermore, the content of Cr was higher in the inner part of the coatings and in the DZ. The results of EDS mapping, see Figure 6, and the quantitative elemental point analyses in Table 1, showed that the outer layer is Ni-Co-Si rich, the intermediate layer of the coating is Cr-Fe-B rich and finally, the DZ is Cr-B enriched (boronized-CoCrFeNiHf_0.1_). In addition, the presence of these phases was also confirmed by EDS point analysis (Table 1), which did not completely match with the expected phases’ chemical composition, since HEAs are complex and have a multicomponent matrix that forms solid solutions, including ordered solid solutions. Thus, the formed phases in the layer are probably mixtures of several elements.

**Figure 9:**
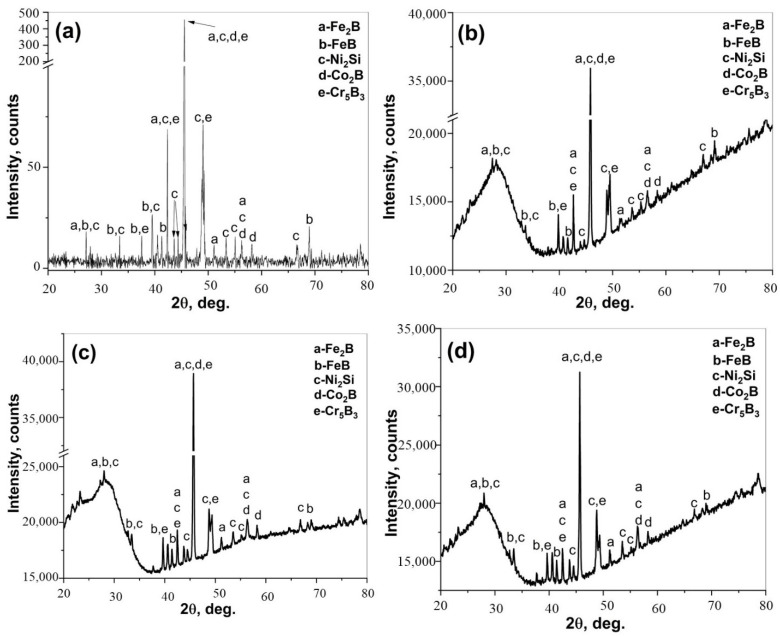
XRD patterns of the borided surfaces; (**a**) CoCrFeNi, (**b**) CoCrFeNiHf_0.1_, (**c**) CoCrFeNiHf_0.2_, and (**d**) CoCrFeNiHf_0.42_.

XRD spectra obtained from the surface after boronizing of CoCrFeNi, and the CoCrFeNiHf_0.1–0.42_ alloys are presented in Figure 9. The XRD analyses demonstrated that SLs + BLs on boronized-CoCrFeNi, and CoCrFeNiHf_0.1–0.42_ contain Ni_2_Si (PDF#00-048-1339), FeB (PDF#01-076-0092), Fe_2_B (PDF#01-072-1301), Co_2_B (PDF#01-089-1994), and Cr_5_B_3_ (PDF#00-032-0278) phases.

## 4. Discussion

In this study, it was found that Hf has a significant effect on the coating morphology, thickness, and microstructure of the BL of CoCrFeNiHf_x_ (x = 0.1, 0.2, and 0.42) alloys.

The regions SL-1, BL, SL-2, and DZ were identified in the borided CoCrFeNi alloys using SEM examination. In the borided CoCrFeNiHf_x_ alloys, three of these regions, SL-1, BL, and DZ were also recognized. The coatings contained Ni_2_Si, FeB, Fe_2_B, Co_2_B, and Cr_5_B_3_ phases for all substrates according to the XRD results. The surface treatment used Ekabor powder, which consists of fine powders of SiC, B_4_C, and KBF_4_ and is used in most common powder pack boronizing processes. It has been reported in the literature that some chemical reactions occur during the boronizing process (Equations (1)–(6)) [37]. The activator agent KBF_4_ initiates the decomposition process and activates the source of free B and Si atoms (Equation (6)) [37,49]. Elemental Si is formed during the reactions of the boronizing process and reacts with Ni at the surface of the alloys. Hence, a combination of boronizing and siliciding processes takes place during the treatment. A correlation between Si diffusion and Ni content has been reported for iron and nickel-based materials. With increasing Ni concentration over a limit of 20% in steel, a Si enrichment occurs in the surface region [22,50]. The concentration of Ni increased at the surface of the alloy, while at the same time Si (released via the reaction of Equation (6)) enrichment occurred at the surface. A Ni-Si rich layer (SL-1) was formed at the surface of the CoCrFeNi alloy (Figure 3 and Figure S1) and this layer can also be observed in Ni-based or Ni-rich alloys [46,50].

KBF_4_ => BF_3_ + KF (1)

2BF_3_ => 2BF_2_ + F_2_ (2)

6F_2_ + B_4_C => 4BF_3_ + C (3)

3BF_2_ => B + 2BF_3_ (4)

2M + B => M_2_B and/or M + B => MB (5)

B_4_C + 3SiC + 3O_2_ => 4B + 2Si + SiO_2_ + 4CO (6)

Boron atoms can diffuse easily into the surface region of the alloy due to their relatively small size and high mobility [46]. In the early stage, B atoms penetrate into the alloy and then react with alloying elements (Equation (5)). Figure 4 shows that the layer thickness of the SL-1 + BL of CoCrFeNi and CoCrFeNiHf_x_ decreases according to their Hf content. The thickness of the SL-1 + BL was approximately 72 µm for CoCrFeNi (Figure 4), which is thinner than for Ni-free steel [30,31,32,33,34,35,36,37], presumably because Ni has strong bonding with Si. Thus, the more the Ni content increased, the higher was the fraction of Ni-Si in the SL-1 + BL, as well as beneath the BL. Hence, this resulted in a reduction of the effective cross-sectional region for B diffusion due to the Ni-Si barrier. Therefore, the large amount of Ni-Si obstructed the diffusion path of B atoms and slowed down the growth of the BL. Finally, the fast-diffusing B atoms contributed to the formation of the BL and the DZ. It is stated that the dark region in the BL and the precipitates in the DZ are Cr-rich boride precipitates [19]. The thickness and microstructure of the SL-1 + BL of the CoCrFeNiHf_0.1_ alloy were similar to the borided layer of the CoCrFeNi alloy. However, when high amounts of Hf were added, the thickness of the SL-1 + BL decreased, whereas the thickness of the DZ increased. Hf was also shown to be effective in determining the morphology and microstructure of the borided layers and the DZs (Figure 3 and Figure 4). Previous studies have shown that Cr, Si, V, Mo, W, and Ti reduce the BL thickness and flatten out the saw-tooth shape of the BL in iron-based alloys [29,30,31,32,33,34,37]. While B penetrated into the alloy and then reacted with alloying elements (Equation (5)), this resulted in the formation of complex boride phases in the substrate (below the BL). Therefore, it can be concluded that the BL prevented the diffusion of Si atoms inwards into the substrate. High concentrations of Fe and Cr were detected in the DZ, which implies the formation of Fe-Cr boride structures (Figure 3).

SiC and B_4_C can react with KBF_4_ and thereby decompose to release Si and B atoms during the boronizing process. The result of these reactions is the base for the siliciding and boronizing processes. The outer part of the coatings on the borided alloys consisted of Co, Fe, Ni, and Si. In the early stage, B atoms penetrate into the alloy and then react with alloying elements [29,30,31,32,33,34,37]. Similarly, Si atoms can diffuse into the surface of alloys and it initially reacts with Ni during the heat treatment processes. Preferential formation of the Ni_2_Si phase was observed. The formation of the Ni-Si phase is not only related to enthalpy and Gibbs energy, but also to the activities of the elements and the chemical potential [19,22].

Both B and Si diffused into the substrates, and the SEM/EDS point analysis and EDS mapping showed that the Si-rich phases were almost completely insoluble in the BL. Si enrichment in the different regions of the BL was also found in the alloys (Figure 3 and Figure 5, Figure 6, Figure 7 and Figure 8). Similarly, while Si enriched regions in the BL were detected in CoCrFeNi, a Si enriched layer in the SL-1 + BL was indicated with elemental analysis (Figure 3 and Figure 6, Figure 7 and Figure 8 and Table 1). Therefore, Si atoms were accumulated close to the BL and they were not transferred deeper into the CoCrFeNi substrate. The solubility of Si was found to be almost zero in iron boride and other metal boride phases (Cr-B). Hence, it can be understood that the BL prevented the transport of Si atoms. However, added Hf in CoCrFeNi alloys could provide transfer of Si atoms into the substrates, but this is happening to a limited extent (see Si maps in Figure 6, Figure 7 and Figure 8).

The thickness of the BL formed on the surface of the CoCrFeNiHf_x_ samples was smaller than for steel and other pure metals under similar conditions due to slower diffusion. CoCrFeNi alloys mainly consist of a random-order solid solution FCC structure [19]. It is suggested that the diffusion in HEAs is slow and that the activation energy is high due to the large and deep fluctuations of the lattice potential between the crystal lattice sites [51]. In addition, the jumping of neighboring atoms into each other’s sites or into vacancies is different than for conventional alloys. It has been noted that the different local configurations of atoms result in different bonding types and different lattice potential energy [52]. The situation is slightly different for alloys with Hf added and the amount of Laves phase in the alloys also plays a role. Hf atoms were doped into the CoCrFeNiHf_x_, which gradually changed the microstructure (Figure 1 and Figure 2 and Table S1). Firstly, the Hf resulted in the formation of Ni-Hf rich phases (Ni_7_Hf_2_) and Si penetrated into the Ni-rich area. On the other hand, Ni-Hf rich phases can serve as the channel or passageway for Si to diffuse inwards. Finally, the increase of the amount of Hf in the alloys changed the diffusion of Si and the thickness of the SL-1 + BL decreased (Figure 4). Furthermore, the thickness of the DZ increased with increasing Hf content (Figure 4). The Laves phase at the SL-1 + BL interface could hinder the inward diffusion of active Si atoms and the amount of the Laves phase increased with a decrease of the Si penetration depth (Figure 6, Figure 7 and Figure 8).

It is clear that CoCrFeNi alloys mainly consist of random-order solid solution with an FCC structure and the diffusion rate is slower than for conventional alloys. The CoCrFeNiHf_x_ alloys consisted of two phases that did not show a random-order solid solution. The CoCrFeNi alloy has coarse grains and therefore the bulk diffusion is dominant. However, the CoCrFeNiHf_x_ alloys are multiphase systems, which have fine grains and also contain eutectic structures. The fine grains and the eutectic structure create a lot of boundaries in the alloys. Hence, grain and phase boundary diffusion are dominant in alloys with added Hf during the boronizing process. In addition, the eutectic structure shortens the diffusion distance needed for the atoms. The eutectic microstructure has a lamellar structure and the distance between these lamellae is very short, about 0.01 µm. Therefore, B diffusion can accelerate considerably in the CoCrFeNiHf_x_ alloys (Figure 3, Figure 4, Figure 5, Figure 6, Figure 7 and Figure 8) and the thickness of the DZ increased with added Hf. However, the penetration distance of Si decreased in the alloy containing the highest amount of Hf (x = 0.42). B is an interstitial atom in these HEAs, and it has high mobility inside the crystal structure. Very fast B movement caused a rapid reaction with Cr (or other elements). The Cr-B rich phase at the SL + BL interface hinders the inward diffusion of active Si atoms and decreases the Si penetration depth.

In summary, Hf plays two important roles in these alloys. Firstly, Hf results in the formation of Ni-Hf rich Laves phase and fine eutectic structures. Secondly, in the coating treatment process, while Si penetration occurred through the Ni-Hf rich phases (Ni_7_Hf_2_ and C15 Laves), B diffusion was easy into the FCC phase regions in CoCrFeNiHf_0.1–0.2_. Furthermore, by increasing the amount of Hf in CoCrFeNiHf_x_, B penetration into the alloys increased due to the fine eutectic structures. Finally, the diffusion of B is faster than that of Si and Cr-B rich areas limiting the transition of Si into the CoCrFeNiHf_0.42_ substrates.

The chemical composition and crystal structure of HEAs are more complex than steels. Therefore, more, and complex, boride phases were formed on the surface of HEAs. The presence of these phases was also confirmed by EDS point analysis and mapping of the elemental distributions (Table 1 and Figure 6, Figure 7 and Figure 8), which did not completely match with these phases’ chemical composition, since HEAs are complex and have a multicomponent matrix and form solid solutions including random-order solid solutions. In addition to this, it can be expected that the alloying elements in HEAs can lead to the formation of mixed boride phases in the coating. Boronizing of Fe-Mo alloys caused the formation of an Fe-Mo-B phase in the layer and the boronizing of Fe-W alloys resulted in a BL and a DZ containing precipitates of Fe-W-B phases [32,37]. High concentrations of alloying elements (Cr, Co, Fe) were detected in the BL that was formed, indicating the formation of binary Fe-B, Cr-B, Co-B, and/or complex ternary Fe-Cr-B and Fe-Co-B (Table 1 and Figure 6, Figure 7 and Figure 8). Table 2 shows the enthalpies and Gibbs free energy of formation for selected phases. However, the reaction rates of Si and B atoms are controlled by the formation energy of the phases (Table 2). Thermodynamically, the formation of a compound or a phase is related to enthalpy and Gibbs free energy, which leads to the formation of the specific compound and/or phase. During the process, the temperature gradually increased from RT to 1000 °C, and B and Si atoms came from the boride powder to the surface of the substrates. The surface of the substrates had a high B and Si concentration during the process. Then, B and Si atoms penetrate into the alloys and then they reacted with the alloying elements of the HEAs. As can be seen from Table 2, the formation of Hf-B and Ni-B is easier than for other compounds and/or phases. The Gibbs free energies of formation of phases are negative and the formation values of Hf-B and Ni-B are more negative than for the other compounds and/or phases in Table 2. This indicates that HfB_2_ and Ni_4_B_3_ are the preferred phases to be formed (Table 2). However, the formation of other phases (Fe-B, Cr-B, Co-B, Ni-Si) was easier during the surface treatment process. Nimonic 90 has been modified using Si and B and in that case, a multilayer coating, consisting of an outer layer of (Ni,Co)_2_Si, an intermediate layer of (Cr,Co)_2_B, and an inner layer of Ti-rich nickel silicide, was observed [46]. It is known that the free Gibbs energy is effective in predicting the occurrence of reactions. Nevertheless, this alone is not enough. The reactions that take place depend on the activity and the chemical potential of the components [53]. The Ekabor powder consists of a Si-rich source and the results revealed that the Si-rich phase was formed in the Si-rich outer region of the alloys. In addition, Ni, Fe, Cr, and Co are abundant in the HEAs.

**Figure 10:**
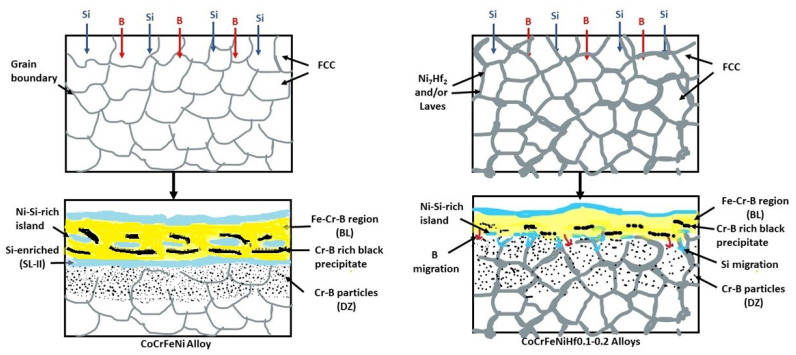
Schematic views of the boronizing mechanism, for CoCrFeNi and CoCrFeNiHf_0.1–0.2_ alloys.

This paper has shown the effect of Hf additions to CoCrFeNi alloys on the boronizing behavior. The effect of Hf is summarized in the schematical model of the boronizing process in Figure 10, and the two mechanisms of boronizing and siliciding are defined. The mechanism is mainly based on the rate of diffusion of B and Si atoms into the substrate to form metal borides and nickel silicides. The B and Si atoms can diffuse easily into the surface of the alloys due to their relatively small size and their diffusion was very fast at the high temperatures (the surface treatment process). In the early stage, B and Si atoms penetrate into the alloys and then they reacted with the alloying elements (Co, Cr, Fe, Ni, Hf), see Figure 10. Therefore, the complex boride phases were formed at the surface of the HEAs. Si atoms were accumulated close to the BL and the DZ and they were not transported into the CoCrFeNi alloy. The Cr-B rich phase at the SL + BL interface would hinder the inward diffusion of active Si atoms and thereby decrease the Si penetration thickness. The added Hf results in the formation of the Ni-Hf phases (Ni_7_Hf_2_/Laves), which may facilitate and become the channel for Si to diffuse inwards.

## 5. Conclusions

The aim of this study was to investigate the effect of the content of Hf on the boronizing and siliciding behavior of CoCrFeNi alloys. For this purpose, we examined the behavior of CoCrFeNi, and CoCrFeNiHf_x_ (x = 0.1, 0.2, and 0.42) alloys after a boronizing/siliciding process.

- Si atoms were accumulated close to the BL and they were not transferred into the substrates, because the BL prevented the transition of Si atoms for CoCrFeNi alloy. With the addition of Hf, Si atoms were diffusing through the Ni/Ni-Hf rich phase into the substrates.
- The thickness of the SL-1 + BL gradually decreased with the addition of Hf, whereas the thickness of the DZ increased.
- Ni_2_Si, FeB, Fe_2_B, Co_2_B, and Cr_5_B_3_ phases were detected in the CoCrFeNi and CoCrFeNiHf_x_ alloys.
- Cr-B precipitates were observed in the BL and DZ of CoCrFeNi and CoCrFeNiHf_0.1_. In CoCrFeNiHf_0.2_ and CoCrFeNiHf_0.42_, Cr-B precipitates were only detected in the DZ. A Cr-B layer had formed between the region of the substrate and the coating for CoCrFeNiHf_0.2_ and CoCrFeNiHf_0.42_. The Cr-B phase was predominantly found in FCC regions of CoCrFeNiHf_0.1–0.2_.
- CoCrFeNiHf_x_ alloys are multiphase systems, which have fine lamellar and eutectic structures. The eutectic structures are dominant when large amounts of Hf are added and the diffusion of B atoms is fast during the boronizing process.

## Figures and Tables

**Table 1:** The chemical compositions obtained with EDS from the points marked in Figure 3.

Alloys	Points/At. %	B K	Si K	Cr K	Fe K	Co K	Ni K	Hf L
CrCoFeNi	1	55	12.8	0.2	1.4	6.3	24.8	-
	2	67	-	0.7	15.6	13.3	2.6	-
	3	63	-	14.6	13.2	7.8	1.4	-
	4	66	-	31.1	2.1	0.4	0.2	-
	5	54	11	2	6.2	10	16.5	-
	6	65	-	17.1	11.1	6.1	1	-
	7	61	-	30.6	4.8	2.7	1	-
CrCoFeNiHf_0.1_	1	30	20.1	-	2	11	37.4	-
	2	20	-	0.3	33	37	9.7	-
	3	46	0.3	0.7	28	21	3.6	-
	4	46	0.1	8.3	23.2	17.5	5	-
	5	-	26.7	2	13.2	22	36.1	-
	6	60	-	36	2.8	1	0.2	-
	7	-	-	3.5	5.5	24.4	45	21.6
	8	-	10	25	24	23	18	-
CrCoFeNiHf_0.2_	1	-	38	0.1	2.90	12.5	46.5	-
	2	50	0.1	1.30	23.5	20.3	4.8	-
	3	-	26.4	18.2	9	14	22.2	10.2
	4	21	0.3	27.9	28	18.7	4.5	0.1
	5	-	5.8	11	30.6	28	24.5	0.1
	6	-	-	9.5	19.8	24.8	35.5	10.4

**Table 2:** Enthalpies and Gibbs free energy of formation for selected phases.

Phase	Enthalpy of Formation, ΔH_f_ (kJ/mol)	Gibbs Free Energy of Formation, ΔG_f_ (kJ/mol)	Ref.
Co_2_B	−128.7 (at 1000 K)	−118.4 (at 1000 K)	[54]
CrB_2_	−94.0 (at 1000 K)	−85.5 (at 1000 K)	[54]
FeB	−73.0 (at 1000 K)	−68.0 (at 1000 K)	[54]
Fe_2_B	−77.8 (at 1000 K)	−68.2 (at 1000 K)	[54]
HfB_2_	−334.9 (at 1000 K)	−324.5 (at 1000 K)	[54]
NiB	−102.4 (at 1000 K)	−93.2 (at 1000 K)	[54]
Ni_4_B_3_	−318.8 (at 1000 K)	−283.5 (at 1000 K)	[54]
Ni_2_Si	−46.9 (at 1123 K)	-	[55]

## Data Availability

Data is not available, as it is part of an ongoing study.

## Supplementary Materials

The following supporting information can be downloaded at: https://www.mdpi.com/article/10.3390/ma15062282/s1, Figure S1: Cross-sectional SEM micrographs of borided-CoCrFeNi alloy; Table S1: Composition of the nominal, full screen-measured, and different regions in the substrate CoCrFeNiHf_x_ alloys by EDS (at. %).
