# Supplementary material for: The Effect of Hf Addition on the Boronizing and Siliciding Behavior of CoCrFeNi High Entropy Alloys
Source: Materials (Basel). 2022 Mar 19;15(6):2282. doi: 10.3390/ma15062282 (PMC8954654; doi:10.3390/ma15062282)
Supplement: Supplementary file 1 [file materials-15-02282-s001.zip › materials-1623840-supplementary.pdf]

materials

# Supplementary Materials: The Effect of Hf Addition on the Boronizing and Siliciding Behavior of CoCrFeNi High Entropy Alloys

**Sezgin Cengiz [1,2,*] and Mattias Thuvander [2,*]**

1 Department of Materials Science and Engineering, Gebze Technical University, Gebze, Kocaeli 41400, Turkey
2 Department of Physics, Chalmers University of Technology, 412 96 Göteborg, Sweden
* Correspondence: sezginc@chalmers.se (S.C.); mattias.thuvander@chalmers.se (M.T.)

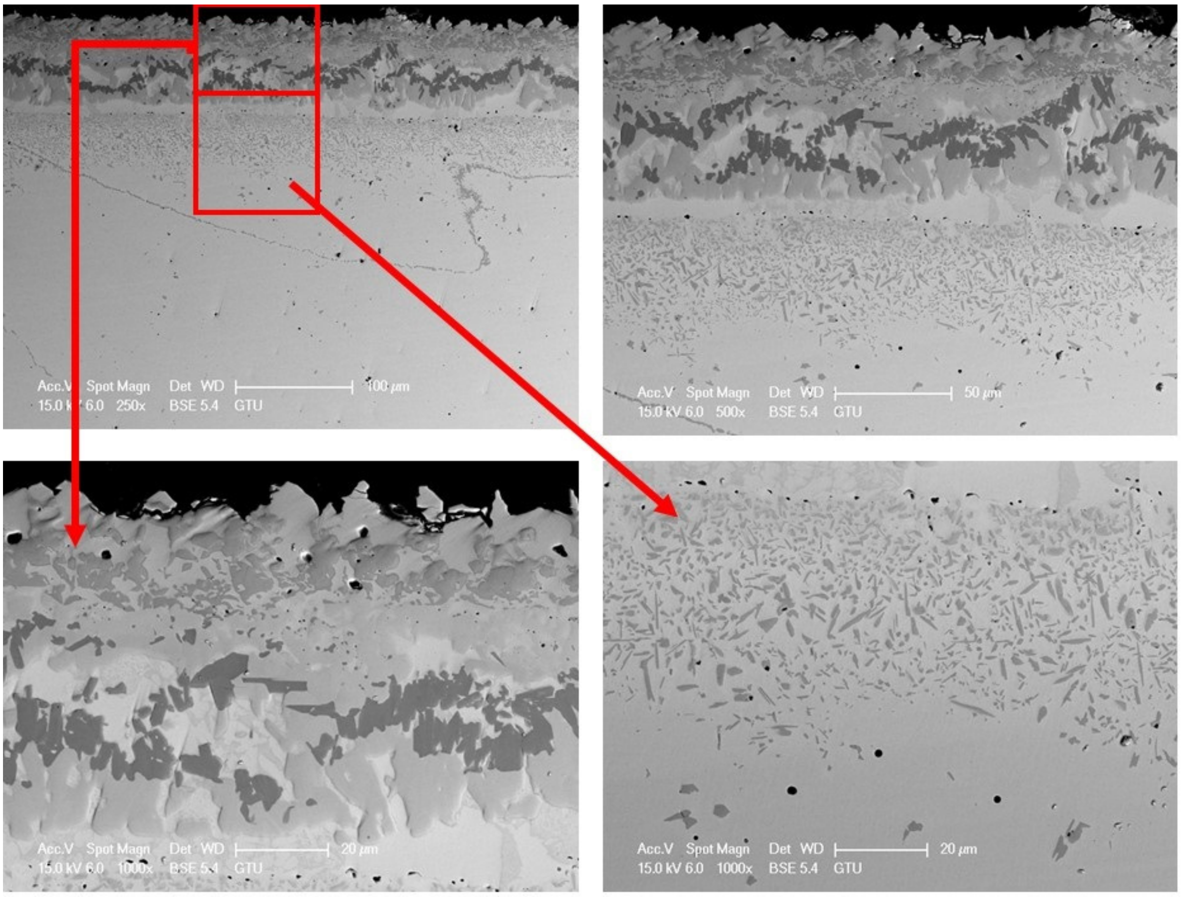


**Figure S1.** Cross-sectional SEM micrographs of borided-CoCrFeNi alloy.

The targeted and the determined compositions by means of EDS of the alloys are given in Table S1. Furthermore, the chemical composition of the FCC, $Ni_7Hf_2$ and Ni7Hf2/Laves phases are measured by using EDS are given Table S1.

**Table S1.** Composition of the nominal, full screen-measured, and different regions in the substrate $CoCrFeNiHf_x$ alloys by EDS (at. %).

| Alloys | Regions | Cr K | Fe K | Co K | Ni K | Hf L |
|---|---|---|---|---|---|---|
| CoCrFeNi | Nominal | 25 | 25 | 25 | 25 | - |
| | Full-screen | 24.53 | 25.46 | 25.53 | 24.48 | - |
| $CoCrFeNiHf_{0.1}$ | Nominal | 24.39 | 24.39 | 24.39 | 24.39 | 2.44 |
| | Full-screen | 25.12 | 24.67 | 24.28 | 23.80 | 2.10 |
| | FCC | 27.05 | 26.34 | 24.64 | 21.80 | 0.18 |
| | $Ni_7Hf_2$ | 10.06 | 12.51 | 20.09 | 41.44 | 15.91 |
| $CoCrFeNiHf_{0.2}$ | Nominal | 23.80 | 23.80 | 23.80 | 23.80 | 4.80 |
| | Full-screen | 24.66 | 24.25 | 23.62 | 23.09 | 4.38 |
| | FCC | 28.65 | 27.63 | 24.03 | 19.69 | - |
| | $Ni_7Hf_2$ + Laves | 9.51 | 19.79 | 24.76 | 35.55 | 10.39 |
| $CoCrFeNiHf_{0.42}$ | Nominal | 22.62 | 22.62 | 22.62 | 22.62 | 9.52 |
| | Full-screen | 22.22 | 22.57 | 23.04 | 22.22 | 9.93 |
| | FCC | 29.57 | 20.57 | 20.83 | 19.32 | 9.91 |
| | Laves | 25.80 | 19.70 | 21.25 | 22.09 | 11.16 |

© 2020 by the authors. Submitted for possible open access publication under the terms and conditions of the Creative Commons Attribution (CC BY) license (http://creativecommons.org/licenses/by/4.0/).
